# Personal and Environmental Challenges of Aging in Place with Long-Term Mobility Disabilities

**DOI:** 10.1093/geroni/igab046.3424

**Published:** 2021-12-17

**Authors:** Widya Ramadhani, Wendy Rogers

**Affiliations:** 1 University of Illinois Urbana-Champaign, Urbana, Illinois, United States; 2 University of Illinois Urbana-Champaign, Champaign, Illinois, United States

## Abstract

Maintaining independence while aging in place at home requires support, especially for older adults aging with long-term mobility disabilities. As age-related changes progress, individuals with long-term mobility disabilities experience more challenges engaging with daily living activities (ADLs) and instrumental activities of daily living (IADLs). To understand the activity challenges of these older adults, we analyzed the interview data from the Aging Concerns, Challenges, and Everyday Solution Strategies (ACCESS) study, a comprehensive user needs assessment of 60 older adults who have had mobility disabilities for at least ten years (Koon et al. 2019). We selected interview data that focused on the conduct of ten activities at home: bathing, dressing, moving around, toileting, transferring, doing hobbies, housekeeping, home maintenance, managing diet and nutrition, and caring for others. This archival study used the coding schemes from the ACCESS study that were developed using both conceptual- and data-driven approaches (Koon et al., 2019). The ecological theory of adaptation and aging (Nahemow and Lawton, 1973) was the underlying framework to identify the challenges related to older adults' functional capacity (personal) and physical environmental barriers (environmental). We identified five main challenges: physical strength, general health limitations, mobility limitations, physical access, and transferring. Older adults' responses to overcoming the challenges involved personal, environmental, and person-environment interaction strategies. This study provides insights into the relationship between the source of environmental barriers and personal coping strategies to guide the design of appropriate aging in place supports for older adults with mobility disabilities.

